# The RootScope: a simple high-throughput screening system for quantitating gene expression dynamics in plant roots

**DOI:** 10.1186/1471-2229-13-158

**Published:** 2013-10-12

**Authors:** Erin J Kast, Minh-Duyen T Nguyen, Rosalie E Lawrence, Christina Rabeler, Nicholas J Kaplinsky

**Affiliations:** 1 Department of Biology, Swarthmore College, Swarthmore, PA, 19081, USA

**Keywords:** Heat shock, Thermometer, Thermostat, Automated imaging, Quantitative imaging, Temperature, Arabidopsis

## Abstract

**Background:**

High temperature stress responses are vital for plant survival. The mechanisms that plants use to sense high temperatures are only partially understood and involve multiple sensing and signaling pathways. Here we describe the development of the RootScope, an automated microscopy system for quantitating heat shock responses in plant roots.

**Results:**

The promoter of *Hsp17.6* was used to build a *Hsp17.6*_p_:GFP transcriptional reporter that is induced by heat shock in Arabidopsis. An automated fluorescence microscopy system which enables multiple roots to be imaged in rapid succession was used to quantitate *Hsp17.6*_p_:GFP response dynamics. *Hsp17.6*_p_:GFP signal increased with temperature increases from 28°C to 37°C. At 40°C the kinetics and localization of the response are markedly different from those at 37°C. This suggests that different mechanisms mediate heat shock responses above and below 37°C. Finally, we demonstrate that *Hsp17.6*_p_:GFP expression exhibits wave like dynamics in growing roots.

**Conclusions:**

The RootScope system is a simple and powerful platform for investigating the heat shock response in plants.

## Background

A systems level understanding of plant responses to the environment requires quantitative gene expression data with high spatial and temporal resolution from multiple individuals [1,2]. This type of data cannot be readily obtained using conventional gene expression assays. RT-PCR, microarray, and RNAseq approaches are limited by low spatial resolution unless tissues are either dissected manually or sorted using FACS. While *in situ* and GUS reporter based approaches can provide high spatial resolution, they require sacrificing the sampled tissue and thus cannot be used to easily monitor gene expression dynamics in individual plants over time. Both Luciferase and fluorescent protein (FP) based reporters expressed in living tissues can provide dynamic expression information, but only FP based reporters allow for both high spatial and temporal resolution.

Several systems for assaying gene expression dynamics in plant roots using FP reporters have previously been developed. All imaging approaches involve tradeoffs between spatial resolution, temporal resolution, and the number of plants that can be imaged at a time. Cost and ease of use are also important considerations. Light sheet microscopy has been used to generate high resolution 3D expression data in an Arabidopsis root at 10 minute intervals, but only one root can be imaged at a time [3]. Two systems have been developed for imaging FPs in multiple roots in rapid succession. The RootChip is a microfluidics based system which allows extended imaging of eight (and potentially more) roots and allows rapid exchanges of the growth media [4,5]. A second microfluidics device, the RootArray, uses confocal microscopy to image up to 64 roots in three dimensions and at high resolution. However, long acquisition times in this system limit the temporal resolution; imaging even a subset of the roots at high resolution takes several hours [6]. While extremely powerful, both of these systems require plants to be grown in the microfluidic device so that the roots grow close to a cover slip which enables high resolution imaging. This is a labor-intensive procedure and requires specialized and relatively expensive microfluidics devices. We are interested in using gene expression dynamics as a phenotype in a genetic screen for high temperature stress 'thermostat’ genes. Because the screen involves characterizing large numbers of individuals we wanted to develop a system which maximizes the number of plants imaged simultaneously while retaining high spatial and temporal resolution and providing accurate control of plant temperature.

The motivation for this screen is to understand the genetic network(s) involved in heat shock (HS) sensing and signal transduction. Plants are sessile organisms and must endure widely varying environmental conditions, many of which result in stress and reductions in yield in agricultural systems. Heat is an abiotic stress that disrupts protein and membrane homeostasis at a cellular level. Heat stress is often accompanied by drought stress which exacerbates its deleterious effects. Global climate change is predicted to result in more frequent and severe episodes of both of these stresses [7,8]. Plants have evolved a complex set of mechanisms for dealing with both stressful as well as non-stressful high temperatures [9-12]. At stressful temperatures the heat shock response (HSR) results in global changes in gene regulation. An important component of the HSR is the synthesis of Heat Shock Proteins (HSPs) which protect normal protein and membrane function. HSP function has been extensively characterized biochemically and genetically and is reasonably well understood [11,13]. Much less is known about the high temperature sensing and signal transduction mechanisms, the 'thermostat’, which regulates the HSR [9].

In Arabidopsis (as in all organisms) HSP expression is strongly induced in response to heat stress. HSP regulation is mediated by the activity of a family of temperature and cAMP regulated Ca^2+^ channels (*CNGC2*,*4*, and *6*), a calmodulin (*CAM3*), a kinase (*CBK3)* and a phosphatase (*PP7*) [14-19]. These genes are predicted to function upstream of a family of highly redundant Heat Shock transcription Factors (*HSF*s) which directly regulate much, but not all, HSP expression [20-23]. HSF activity is also thought to be regulated through direct physical interactions with the HSPs HSP70 and HSP90 and a HS induced HSF binding protein (HSBP), creating feedback loops which finely tune the HSR [24-27]. Notably, none of these thermostat genes were identified based on their mutant phenotypes, perhaps due to redundancy in the HSR network. Instead, these genes were identified based on homology with other systems, expression profiling, or biochemical interactions and they have all been characterized using reverse genetics approaches.

Many details of the HS thermostat’s function are complex and not fully understood. The CNGC and HSF gene families exemplify this complexity. Based on studies in mammalian systems, CNGCs are thought to function as hetero-tetramers and the HSFs as homo- and hetero-trimers [14,27,28]. The *in vivo* subunit composition of these complexes has not been described in plants. Since the function of these genes is likely influenced by their co-complex interaction partners the analysis of their mutant phenotypes is complicated. Both the CNGC and HSF families contain members which, when mutated, exhibit opposite phenotypes. *cngc2* and *4* mutants increase Ca^2+^ influx into cells and result in elevated thermotolerance while *cngc6* mutants decrease Ca^2+^ influx and exhibit decreased thermotolerance [14,17]. Similarly, *HSFA1a,b,d*, and *e* are positive regulators of the HSR and are required for thermotolerance while *HSFB1* and *HSFB2b* attenuate the HSR [20,21,23]. Not only are the physical interactions among Arabidopsis family members not well understood, but their interactions with and regulation by other molecules are also poorly described. For instance, the CNGCs are activated by the second messenger cAMP yet only a few genes involved in cAMP production have been identified in Arabidopsis. None have demonstrated biological functions in plants [14,29,30]. Given these ambiguities it is likely that multiple components of the HSR network remain unidentified.

Unbiased forward genetics approaches based on thermotolerance defects have identified only a few HSR genes including HSP101 (*HOT1*), a chitinase (*HOT2*), and an exportin (*HIT2*) [31-33]. None of these genes have obvious sensing or signaling functions and, to our knowledge, no thermostat genes have been identified by a high temperature thermotolerance screen. This suggests that screening for thermotolerance defects using conventional assays may be of limited value in identifying HS thermostat genes.

**Figure 1:**
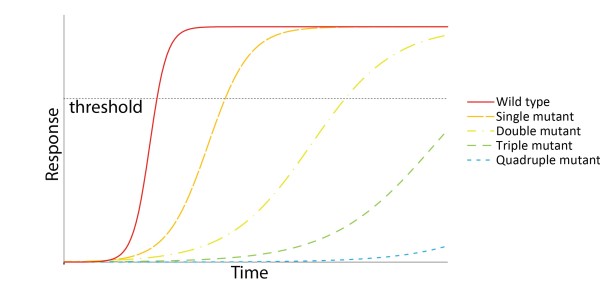
**A model of how overlapping functions in a gene family might obscure thermotolerance phenotypes.** Single and double mutants of the *HSFA1* transcription factors have no thermotolerance phenotypes [21,23], presumably because they still cross a response threshold before the phenotype is assayed. Triple and quadruple mutant phenotypes are detected because they do not cross the threshold. A system that allows the kinetics of the response to be monitored could uncover these single and double mutant phenotypes.

Forward genetic approaches have failed to identify the signaling and sensing components of the HSR. This may be attributed to: 1) redundancy in the network of genes that makes up the thermostat, and 2) phenotyping approaches which assay the end point of the HSR as opposed to its dynamics. Evidence for the masking effects of redundancy and the limitations of conventional phenotyping approaches comes from studies on the HSF transcription factors. To uncover thermotolerance phenotypes due to a loss of *HSFA1* function, at least three family members must be knocked out [21,23]. Similarly, *hsfb1* single mutants have no HSP expression or thermotolerance phenotypes while h*sfb1/hsfb2b* double mutants have both [20]. Figure 1 illustrates why phenotypic assays with detection thresholds, especially assays which take place at a single late time point, are likely to overlook the effects of genes with redundant family members (Figure 1).

To overcome these limitations we have developed the RootScope which generates the quantitative data required to identify new and characterize existing components of the HSR thermostat. The RootScope consists of a robust HSR reporter and an automated imaging system for monitoring the reporter in multiple plants with high temporal and spatial resolution.

## Methods

### Construction of the HSP17.6_p_:GFP reporter line

The Hsp17.6 C-CI (AT1G53540) promoter was amplified from genomic DNA using primers AT1G53540-FWD (5′-CACCATTCAGGTAATCAGGTTGTCTGC-3′) and AT1G53540-REV (5′-CGTTTCACTTCCTCTTGTGATTGC-3′). These primers amplify 713 bp starting in the 3′ UTR of the upstream gene AT1G53530 and ending at the ATG start codon of AT1G53540. CACC was added to the 5′ end of AT1G53540-FWD to provide directionality for TOPO cloning into pENTR/D-TOPO (Invitrogen, Grand Island, NY). TOPO cloning of the PCR product into pENTR/D-TOPO was followed by Gateway mediated LR recombination (Invitrogen) into destination vector pFAST-R07 (Shimada et al., 2010) to create a C-terminal promoter:GFP fusion (HSP17.6_p_:GFP). This construct was transformed into Col-O plants using *Agrobacterium tumefaciens* strain GV3101 (Clough and Bent, 1998). Primary transformants were identified using the RFP seed marker contained in the pFAST-R07 construct and were allowed to self-fertilize to generate homozygous lines. Homozygous lines were screened for GFP expression at basal temperatures (22°C) and after a 1 hour 37°C heat shock as 3 day old seedlings. Fluorescence imaging was performed using a Leica MZ16F microscope equipped with a 2x Planapo lens, a GFP3 filter set, and an EL6000 light source (Leica Microsystems).

### Construction of the automated imaging system

The heated stage consists of an Inheco CPAC Ultraflat HT microplate heater with a temperature range of 4°C to 110°C (Inheco). Microplates were placed on the CPAC and held in place by a custom 3D-printed chamber which incorporates the heated glass plate from a Tokai Hit INU-GSI stage top incubator. For horizontal root growth the heated stage was mounted on an ASI LS-50 linear stage which provided Z (focus) movement. The LS-50 linear stage was then mounted on an ASI MS-2000 XY stage to create a heated XYZ stage. For vertical root growth a heated XY stage was built by directly mounting the heated stage on the MS-2000 XY stage. All ASI stages were controlled by a LX-4000 control unit (ASI).

Images were acquired using a Hamamatsu ORCA II BT-512 camera cooled to -55°C in both horizontal and vertical arrangements. Exposure times ranged from 75–200 msec depending on the plant line being imaged. For horizontal root growth the heated XYZ stage and camera were mounted under a Leica MZ16F microscope equipped with a 2x Planapo lens, a GFP3 filter set, and an EL6000 light source (Leica Microsystems). For vertical root growth the heated XY stage was mounted vertically on a 1.5″ mounting post (Thorlabs). A Leica Z16 APO macroscope with a 2x Planapo lens and a 1x tube lens (Leica) was coupled to a DFM dichroic filter cube with a MDF-GFP filter set (Thorlabs) and the ORCA II camera using custom adapters. Illumination was provided by a M470-L2 high power LED collimated using an ACL2520-A lens (Thorlabs). These components were mounted on a 8″ x 24″ x 1/2″ aluminum breadboard which in turn was mounted on a Parker Daedal CR4900-8 crossed roller bearing stage (Parker Daedal). The LS-50 linear stage was connected to the breadboard to provide focus control by moving the entire microscope relative to the XY stage. All of these components were built up on a 12″ x 36″ optical breadboard (Vere).

Both the LED and EL6000 light sources were shuttered using a USB connected Arduino UNO driving a SainSmart 2-Channel relay (SainSmart). For the EL6000 light source the relay was directly connected to the light source shutter inputs. For the high power LED light source the relay was connected to the DIM input of a Luxdrive 1400 mA Buckblock (LUXdrive) which was used to drive the LED.

μManager microscopy automation software [34] running on a 64 bit Windows 7 (Microsoft) system was used to control the Arduino based shutter, the LX-4000 stage control unit, and the ORCA II camera. Root images were quantitated and, in some cases straightened, using custom ImageJ [35] and Perl scripts. Alignment of straightened roots was performed using the Template Matching and Slice Alignment ImageJ plugin [36].

### Plant growth

Arabidopsis seeds were surface sterilized with 30% bleach and 0.1% SDS for 15 minutes, rinsed three times in sterile water, and plated on OmniTray single well microplates (Thermo Scientific product #242811) containing 60 ml of 0.5x MS media. The plated seeds were stratified for three days at 4°C and then transferred to Percival E-30B growth chambers (Percival Scientific) set at 22°C with 24 hours of light for three days.

## Results

### Generation of a *Hsp17.*6_p_:GFP reporter for the heat shock response

Assaying the HSR in multiple plants with high spatial and temporal resolution requires a live cell reporter with a high dynamic range and a strong signal when induced. We chose *Hsp17.6* (AT1G53540) because this small HSP is consistently among the most highly induced HSPs in both our own and published HS microarray experiments [21,37,38]. We cloned the AT1G53540 promoter upstream of GFP in the pFAST-R07 vector [39] and transformed the resulting *Hsp17.6*_p_:GFP construct into Arabidopsis. We used this vector because it contains a RFP seed marker which allows for easy identification of plants which contain the reporter. This feature will be useful for identifying plants homozygous for the reporter in the future. We selected 46 independent transformants and allowed each to self-fertilize to generate plants homozygous for the reporter. Homozygous lines were screened for GFP expression in 3 day old roots at basal temperatures and an hour after a one hour 37°C heat shock. None of the lines exhibited GFP expression at basal temperatures, consistent with a lack of *Hsp17.6* expression in the absence of cellular stress. The intensity of the GFP signal after the heat shock varied among lines and we chose three of the brightest lines (8, 11, and 26) for further characterization (data not shown).

### A simple high-throughput screening system for quantitating gene expression dynamics in plant roots

**Figure 2:**
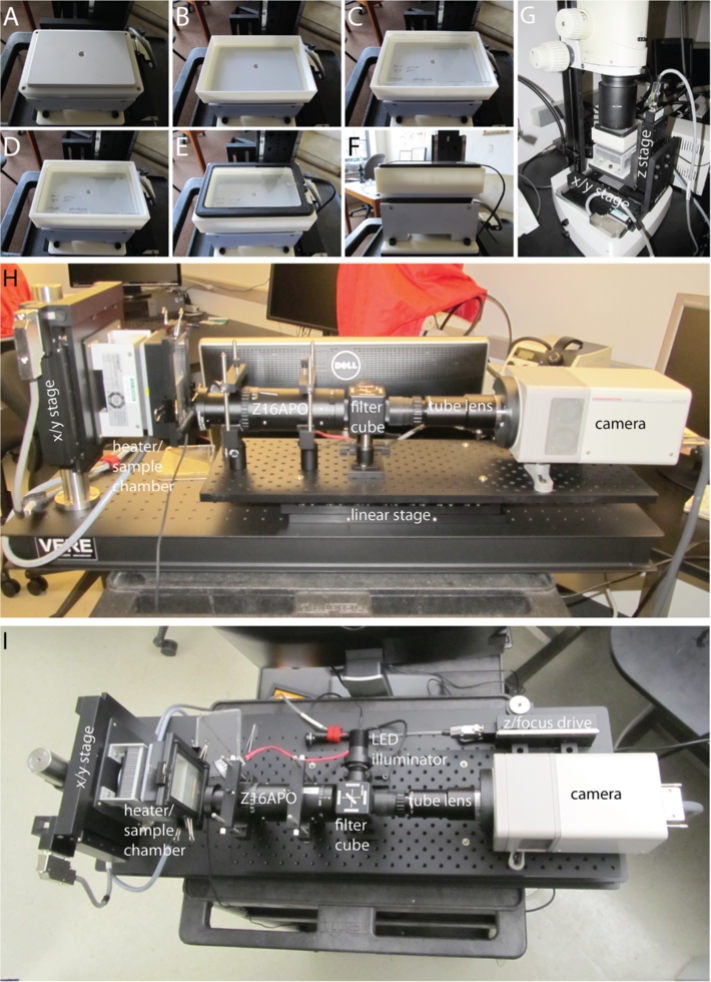
**An overview of RootScope construction.** A heating/cooling block is used to provide temperature control **(A)**. A chamber is placed on the heating/cooling block **(B)** and a microplate containing the plants to be imaged is placed in the chamber **(C)**. A gasket is placed on top of the chamber **(D)** allowing the heated glass plate to sit on top of and seal the chamber **(E)**. The resulting stack of components (side view, **F**) can then be mounted either horizontally **(G)** or vertically **(H**-**I)** on an automated microscope.

To monitor the *Hsp17.*6_p_:GFP reporter we built an automated fluorescence microscopy system controlled by μManager software [34]. The microscope has a temperature-controlled chamber consisting of a Peltier heating/cooling device that accepts a petri dish with the same size format as a 96 well plate. Because condensation on the lid of the petri dish would prevent imaging we incorporated a heated glass lid into the chamber (Figure 2A-F). The chamber was mounted on a motorized XY stage to allow multiple roots to be imaged in rapid succession. The chamber can be used horizontally on the stage of a conventional upright fluorescence microscope (Figure 2G). In this orientation roots grow horizontally across the surface of the media. The chamber can also be mounted vertically on a custom fluorescence microscope (Figure 2H-I), which allows the roots to grow vertically on the surface of the media. Vertical growth is advantageous because, when grown horizontally, about half of the roots stop growing horizontally, attempt to grow into the media, and thus cannot be imaged.

The advantage of this setup compared to a microfluidic device is that we can easily and quickly plate 100 (or more) seeds on MS media on a single, cheap, and commercially available microplate. Seed placement is not critical as the roots do not have to grow in defined micro-channels. Root positions are identified manually using the μManager slide explorer plugin once the plate has been transferred to the microscope for imaging. The seeds are germinated in an incubator, grown for 3 or 4 days, and then transferred to the microscope where they can be simultaneously heat shocked and imaged. The system allows us to quantitate the HSR at high temporal (every 4 minutes with 100 plants) and spatial (~2.4μm with our current camera and optics) resolution with a 12 bit dynamic range.

### Characterization of the induction and attenuation kinetics of the *Hsp17.6*_p_:GFP reporter

**Figure 3:**
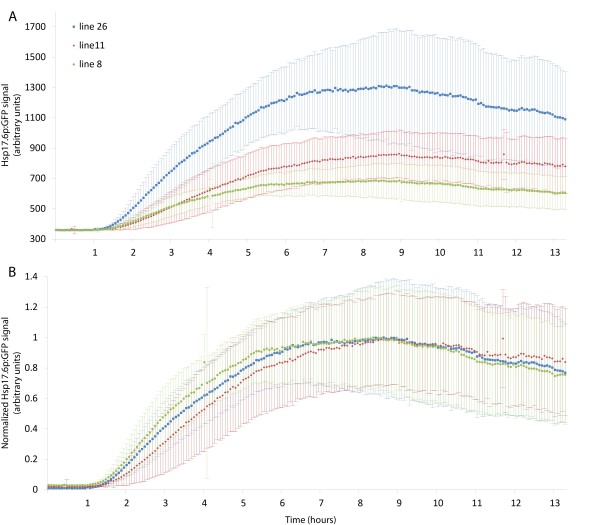
**Comparison of*Hsp17.6***_**p**_**:GFP expression levels and dynamics in three independent transformants.** Four day old seedlings from lines 8 (green), 11 (red), and 26 (blue) homozygous for *Hsp17.6*_p_:GFP were heat shocked at 37°C starting at time point 0 and imaged every four minutes. Induction of the reporter over 13:20 is shown without **(A)** and with normalization **(B)**. Error bars are +/- standard deviation.

Seeds homozygous for *Hsp17.*6_p_:GFP from lines 8, 11, and 26 were plated on the same plate, germinated at 22°C, and at four days placed in the growth chamber on the microscope and heat shocked continuously at 37°C. The roots were imaged every four minutes for 800 minutes. Induction was detectable at one hour and peaked at approximately eight hours in all lines. The intensity of the GFP signal varied between lines; line 26 was the brightest and line 8 was the dimmest (Figure 3A). To determine the variability of the induction kinetics of these three lines we normalized their maximum fluorescence intensity. All three lines exhibit very similar kinetics (Figure 3B) suggesting that line 26, while brighter than the other lines we screened, exhibits similar kinetics to the other lines and is not brightest because of an abnormal expression pattern. Because it has the highest dynamic range and normal induction kinetics, line 26 was used exclusively from this point on and all references to *Hsp17.6*_p_:GFP refer to line 26.

**Figure 4:**
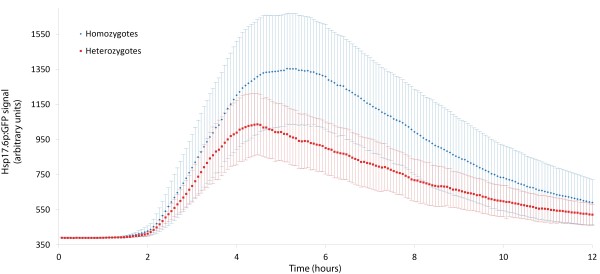
**Induction kinetics of*Hsp17.6***_**p**_**:GFP homozygous and heterozygous plants.** Plants homozygous (blue) and heterozygous (red) for *Hsp17.6*_p_:GFP were heat shocked for two hours at 37°C and imaged every four minutes over a 12:00 period. Error bars are +/- standard deviation.

To characterize the attenuation of *Hsp17.6* expression after a short heat shock we heat shocked *Hsp17.6*_p_:GFP for two hours and then reduced the temperature to 22°C, imaging the plants every 4 minutes. This HS regimen results in the induction of *Hsp17.6*_p_:GFP at approximately one hour (starting half way through the heat shock) and attenuation at five hours. We performed this analysis with plants both homozygous and heterozygous for *Hsp17.6*_p_:GFP to determine if the dosage of the reporter affected the intensity or the kinetics of the reporter. Although the induction kinetics for heterozygotes and homozygotes were similar, plants containing a single copy of the reporter were on average only 62% as bright as plants homozygous for the reporter and attenuation occurred earlier in heterozygotes (Figure 4). This result highlights the importance of ensuring matched copy numbers when using the *Hsp17.6*_p_:GFP reporter to characterize the HSR.

### *Hsp17.6*_p_:GFP expression correlates with the heat shock temperature

**Figure 5:**
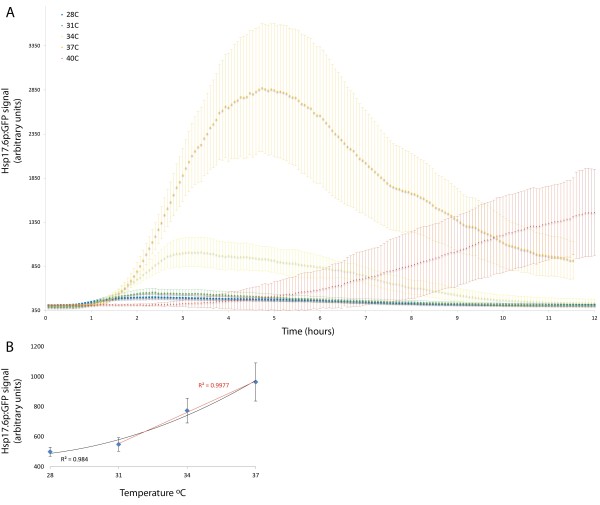
**Induction kinetics of*Hsp17.6***_**p**_**:GFP in response to heat shock temperatures from 28°C to 40°C.** Plants were heat shocked for one hour at temperatures from 28-40°C and imaged every four minutes for at least 11:30 **(A)**. The average intensity of the GFP signal from time points between 2:00–2:20 (1:00–1:20 after the end of heat shock) are plotted with either a linear regression (31-37°C, red) or a 2° order polynomial regression (28-37°C) **(B)**. Error bars are +/- standard deviation.

To determine if the reporter could be used as a read out of root temperature we exposed plants to heat shock temperatures between 28 and 40°C for one hour. Consistent with existing array data for *Hsp17.6*, very little response was detected below 30°C, a temperature at which Arabidopsis root growth is largely inhibited. The magnitude of the induction is linear from 31–37 degrees (R^2^ = 0.9977) and the response over the temperature range from 28–37 degrees can be modeled with a 2° order polynomial (R^2^ = 0.984). This demonstrates that the reporter can be used to accurately report high temperatures in the root (Figure 5). The maximal response was observed at 37°C, a result similar to those of a previous experiment in which acquired thermotolerance (AT) was maximally induced at 37°C [22]. These data suggest that *Hsp17.6* is induced by a HS thermostat that is distinct from the thermostat which function at non-stressful temperatures (<28°C) to regulate growth and development [12,40].

**Figure 6:**
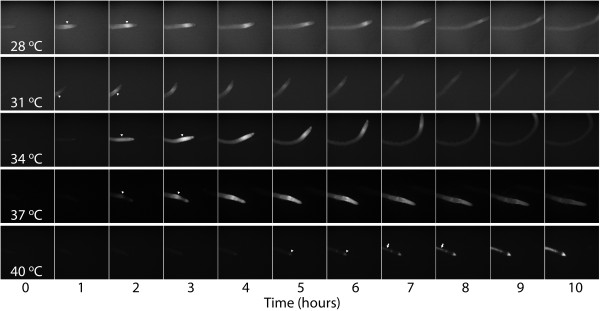
**The location of*Hsp17.6***_**p**_**:GFP expression maxima is temperature dependent.** Hourly images of representative roots heat shocked for an hour at 28-37°C are shown. Arrowheads indicate the location of the brightest signal for the first two time points where the maximum is clear (either the differentiation zone in roots heat shocked from 28-37°C or the root tip in roots heat shocked at 40°C). Arrows indicate late vascular expression in roots heat shocked at 40°C. The contrast of all images in each temperature series was uniformly enhanced using the ImageJ 'enhance contrast’ tool so that both low and high expression patterns can be seen. As a result, intensity should not be compared between temperature series. Quantification of signal intensity can be found in Figure 5.

Both the kinetics and the localization of the *Hsp17.*6_p_:GFP response are markedly different at 40°C compared to lower temperatures. At 40°C induction does not occur until after the heat shock ends, suggesting that at temperatures above 37°C the HSR machinery itself may be inhibited (Figure 5). Again, this result is consistent with the observation that acclimation temperatures above 37°C result in lower levels of AT [22]. In addition to differences in the kinetics of the response, the localization of the response maximum is different below and above 37°C. Below 37°C the response maximum occurs in the differentiation zone at approximately 5 hours. In contrast, at 40°C the maximum signal occurs after 10 hours and is initially located at the root tip and subsequently in the root tip and the vasculature (Figure 6 and Additional file 1). The marked difference in kinetics and localization supports the idea that multiple HS thermostats exist in plants and demonstrates the temporal and spatial resolution of our system.

### *Hsp17.6*_p_:GFP expression exhibits wave like dynamics

**Figure 7:**
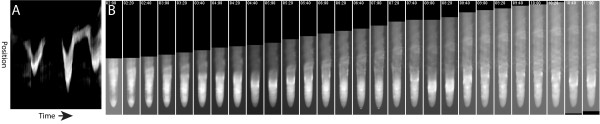
**Oscillating waves of*Hsp17.6***_**p**_**:GFP expression during root growth after a 1 hour 37°C heat shock.** A kymograph display of the position of maximal *Hsp17.6*_p_:GFP expression in the epidermis **(A)**. Images of a straightened and aligned root exhibiting *Hsp17.6*_p_:GFP oscillations at 20 minute intervals **(B)**.

Several thousand genes expressed in the root (3493) have been shown to oscillate in two phases with a periodicity of ~6 hours in the meristematic and elongation zones. These oscillations were characterized at relatively low spatial and temporal resolution using luciferase based markers and microarray data and are associated with developmental events including root bending and branching. The periodicity of these oscillations was stable over a temperature range of 18-24°C, however the effects of stressful high temperatures (>30°C) on these oscillations were not characterized [41]. Plants analyzed in these studies were grown under normal conditions and thus did not express the HSR genes, which are expressed only in response to cellular stress. To determine if *Hsp17.6* expression oscillates we examined the time lapse series from *Hsp17.6*_p_:GFP roots which had been heat shocked for one hour at 37°C. After induction, *Hsp17.6*_p_:GFP exhibited wave like expression dynamics in the epidermis, sweeping from the differentiation zone through the elongation zone to the meristem and back again. The periodicity of these waves was 3.9 +/- 0.8 hours (n = 15). The waves were associated with developmental events, sweeping in front of cell differentiation as visualized by the appearance of root hairs on epidermal cells (Figure 7 and Additional file 2). Because *Hsp17.6* is not expressed at basal temperatures it is unclear whether the differences in periodicity between the previously described genes and *Hsp17.6*_p_:GFP are due to multiple oscillating circuits of gene expression in the root, to the effects of the heat shock on the wavelength of the oscillations, or due to differences between the folding and degradation rates of luciferase and GFP.

## Discussion

The RootScope is a simple and flexible system that makes it possible to determine the dynamics of gene expression in large numbers of plant roots with high temporal and spatial resolution. The time and cost required to set up plants for screening are minimized since seeds are plated on standard media in microplates and plating seeds for an experiment takes only minutes. The most time consuming step in a RootScope experiment is the manual identification of root positions once the plate is transferred to the microscope. This step takes less than 20 minutes per plate for an experienced undergraduate student. It should be possible to automate this task using image recognition algorithms; however, since automated localization may need to be confirmed and fine-tuned manually, the advantages of automating this step may not be significant in practice. In this paper we report using the RootScope as a fluorescence microscope for quantifying a GFP based transcriptional reporter. Similar to existing non-fluorescent phenotyping systems such as Phytomorph, the microscope is flexible and could also be used to generate high resolution incident light images to characterize growth dynamics in multiple plants simultaneously [42,43]. Because the optics we have used are based on a zoom macroscope both larger and smaller fields of view can be selected (with concomitant changes in resolution). Finally, although we have chosen to image roots, there is no technical reason why the aerial portions of plants could not be imaged using this system.

The induction kinetics of *Hsp17.6* in this system is slower than previous reports of HSP protein and transcript accumulation in which HSPs accumulate within half an hour of a heat shock [44,45]. The sensitivity of our system and the maturation kinetics of GFP are both likely to contribute to this difference. Both RT-PCR and enzymatic Western blot detection techniques amplify small signals significantly making it possible to detect small numbers of molecules while our imaging approach does not. This limits the sensitivity of our system at very low *Hsp17.6* expression levels. The folding, chromophore maturation, and degradation dynamics of GFP are also likely to influence the kinetics reported here, most likely by introducing delays in both the appearance and attenuation of the GFP signal. *Hsp17.6*_p_:GFP was built using pFAST-R07 which contains EGFP [39,46]. EGFP is a GFP variant which exhibits approximately a four fold increase in the rate of chromophore maturation compared to wild type GFP. GFP variants with even faster maturation rates such as GFPmut2 or GFPm have been demonstrated to mature over twice as fast as EGFP *in vitro*[47] although no *in planta* maturation rate data exist in the literature. While the use of a rapidly maturing GFP variant has the potential to decrease the delay between transcription and detection of the GFP signal using the RootScope, there is no reporter that we are aware of which can completely eliminate this delay. Since we are not concerned with the absolute timing of these events and given the relatively rapid expression dynamics we have observed, we do not anticipate that these artifacts will prohibit the use of this system for identifying HSR thermostat mutants. In addition to using a rapidly maturing GFP to decrease delays in the onset of the signal, the use of a translational as opposed to a transcriptional reporter might result in more accurate degradation kinetics.

The ability to monitor the kinetics of the HSR using *Hsp17.6*_p_:GFP should allow us to take a forward genetics approach to identifying HSR genes based on changes in *Hsp17.6*_p_:GFP induction and decay kinetics. We have EMS mutagenized 3,000 *Hsp17.6*_p_:GFP seeds and harvested pools of 4–5 self-pollinated plants. 80 seeds from each pool will be screened on plates with 20 non-mutagenized seeds as controls. This pooled approach should allow us to identify most of the mutants in each family and strikes a balance between throughput (each run takes 12 hours) and coverage. Assuming 200 days of screening in the first year we could screen 800–1000 families which, while it would not reach saturation, should uncover several HSR thermostat mutants [48]. We will inspect the data both manually and computationally (based on extracting the times at which each plant’s HSR signal rises above a baseline and reaches a maximum and on the slope of the induction and attenuation phases) to identify mutants that have altered HSR kinetics, localization, or respond as if they are hotter or colder than they actually are. In addition to identifying HSR thermostat mutants we also anticipate discovering mutations which disrupt the wave like expression dynamics of *Hsp17.6*_p_:GFP. These mutants may provide insights into the co-ordination of developmental events and stress responses. This system will also be useful for uncovering the effects of previously identified HSR genes (many of which do not display single mutant phenotypes) on HSR dynamics.

## Conclusions

We have demonstrated that the RootScope can be used to generate gene expression data with high temporal and spatial resolution in multiple plants simultaneously. We have used the system to uncover evidence that supports the hypothesis that multiple distinct high temperature responses exist in plants. Additionally, we have documented previously unknown waves of HS induced gene expression. Apart from characterizing the HSR, the RootScope should be of general use for quantitating other types of gene expression dynamics. Using other types of reporters [2,49], it should also be useful for quantitating metabolite and small molecule dynamics in plant roots.

## Competing interests

The authors declare that they have no competing interests.

## Authors’ contributions

NK, EK, DN, and RL designed experiments. NK built the automated microscope. RL and CR built the *Hsp17.*6_p_:GFP reporter and transformed it into plants. DN and CR characterized transformed plant lines and generated homozygous stocks. NK, EK, and DN performed and analyzed RootScope experiments. All authors helped to draft and approved the final manuscript.

## Supplementary Material

Additional file 1 **Induction kinetics of *Hsp17.6***_**p**_**:GFP in response to heat shock temperatures at 37°C and 40°C.** Roots heat shocked for one hour at either 37°C (top) or 40°C (bottom) were imaged every four minutes for 11 hours. Click here for file

Additional file 2 **Oscillating waves of epidermal *Hsp17.6***_**p**_**:GFP expression during root growth after a 1 hour 37°C heat shock.** Roots were heat shocked for one hour and imaged every four minutes for 11 hours. Root images were computationally straightened and aligned to allow analysis of the position of the wave. Click here for file
